# DMFRNet: Dynamic Multi-Scale Feature Reweighting Network for Dairy Cow Detection

**DOI:** 10.3390/ani16162520

**Published:** 2026-08-12

**Authors:** Zhihang Wei, Gaohan Zhao, Zhiyu Yu, Xiaoqian Li, Qiuchen Li, Donghui Wei

**Affiliations:** 1 College of Intelligence Science and Engineering, Northeast Agricultural University, Harbin 150030, China; s241401023@neau.edu.cn (Z.W.); s2514012025@neau.edu.cn (G.Z.); s241402064@neau.edu.cn (Z.Y.); s241402051@neau.edu.cn (Q.L.); 2 College of Agriculture, Northeast Agricultural University, Harbin 150030, China; s2503022046@neau.edu.cn

**Keywords:** dairy cow, object detection, smart livestock farming, attention mechanism

## Abstract

Achieving accurate cattle detection in complex barn environments is a critical technical challenge for smart livestock farming. Cattle exhibit highly similar appearances, severe occlusion, and significant multi-scale variations, making it difficult for existing detection methods to balance accuracy with model efficiency. This paper proposes a lightweight cattle detection model, DMFRNet, with YOLO11 as the baseline. To address these challenges, DMFRNet introduces three targeted improvements. First, C3K2-DIMB is designed to enhance multi-scale feature extraction by adaptively reweighting multi-branch depthwise convolution features, thereby improving the representation of cattle with different body sizes, poses, and viewing distances. Second, SimAM is embedded after the SPPF layer to refine high-level semantic features without introducing additional parameters, which improves feature discrimination under occlusion, low contrast, and complex backgrounds. Third, LSCDH replaces the original decoupled detection head to reduce parameter redundancy through cross-scale shared convolution while preserving multi-scale prediction capability. These designs jointly address the key challenges of multi-scale cattle appearance, occlusion, and lightweight model construction in complex barn scenes. Experimental results on the combined CBVD-5 and Dairy Cow dataset demonstrate that DMFRNet achieves a Precision of 93.49%, F1 of 89.84%, mAP50 of 93.85%, and mAP50–95 of 61.74%, with only 2.16 M parameters, 5.10 GFLOPs, and a model size of 4.5 MB. Comparative experiments demonstrate that DMFRNet provides a favorable accuracy–efficiency trade-off for lightweight dairy cow detection.

## Simple Summary

Accurate dairy cow detection is important for automatic barn monitoring, but it remains challenging because cattle often appear at different scales, overlap with each other, and are captured under uneven illumination. The model addresses three practical challenges: dynamic multi-scale feature extraction is used to represent cattle with different body sizes and poses, parameter-free attention is introduced to enhance discriminative features under occlusion and low-contrast conditions, and a lightweight shared detection head is designed to reduce model complexity. Experimental results show that DMFRNet improves localization accuracy while maintaining a compact model size. This study provides an efficient detection approach for dairy cow monitoring in complex farming environments.

## 1. Introduction

Cattle farming is one of the core pillar industries of modern animal husbandry, and the health status and behavioral patterns of cattle directly affect the yield, quality, and economic returns of livestock products. In recent years, artificial intelligence (AI) and Internet of Things (IoT) technologies have been increasingly adopted in agriculture, and intelligent livestock farming has become an important direction in modern animal husbandry [1,2]. Traditional manual inspection methods require considerable effort and are prone to observer bias, making continuous monitoring infeasible in large-scale farms [3,4]. Dairy cow behaviors in barns are inherently complex: drinking, feeding, lying, and walking routinely occur under mutual occlusion, varying illumination, and substantial body size differences, all of which significantly increase detection difficulty [5,6,7]. Recent progress in computer vision and deep learning has provided new methods for intelligent livestock monitoring [8,9,10].

Object detection approaches are generally divided into two-stage and single-stage algorithms. Two-stage methods such as R-CNN [11] and Fast R-CNN [12] deliver high accuracy but incur heavy computational cost, whereas single-stage detectors such as SSD [13] and YOLO [14] offer a more favorable trade-off between accuracy and speed. Consequently, improved methods built upon the YOLO series have become a research hotspot. In recent years, researchers have enhanced model performance through attention mechanisms and network architecture optimization across diverse livestock and wildlife monitoring tasks. Rahman et al. developed an IoT-based intelligent aquaculture system integrating ESP32, Jetson Nano, and YOLOv4-Tiny for fish detection, achieving a strong balance between performance and computing cost [15]. Li et al. proposed WildMDT-YOLO, a YOLOv8n-based wildlife detection method combining multi-scale feature modules, DCNv3, and hybrid attention to improve feature learning, which improved mAP by 3.1% over the baseline while maintaining robust small-object and cross-dataset performance [16]. In the livestock domain, Chao et al. presented DGS-YOLO for pig face recognition, incorporating dynamic convolution, C3k2_GBC modules, SimAM attention, and Shape-IoU loss to improve accuracy, recall, and mAP50 by 4%, 2.1%, and 2.3%, respectively, with enhanced generalization in small-sample scenarios [17]. Bai et al. introduced SAMS-KLA-YOLO11 for sheep face detection and keypoint localization, integrating SAMSConv for multi-scale feature extraction, KLAttention for efficient feature refinement, and EIoU loss, achieving better precision, recall, and mAP than standard YOLO models while reducing model size and complexity [18]. Mar et al. proposed a video-based cow detection and tracking system that combined instance segmentation with location, color, texture, and CNN-based features for multi-object tracking, demonstrating the usefulness of multi-feature visual information for continuous cattle monitoring [19]. Jiang et al. proposed a real-time poultry monitoring framework built on an attention-enhanced YOLO-Chicken model combined with BoT-SORT tracking, achieving a 1.35% improvement in detection accuracy over the original YOLOv8 while surpassing RetinaNet [20]. Ke et al. introduced IECA-YOLOv7 for small wild animal detection on grasslands, employing an improved channel attention module, CARAFE upsampling, and NWD loss, reaching 86.6% mAP@0.5 with precision and recall improving by 2.9% and 1.8% over YOLOv7-tiny, demonstrating excellent small-object detection and cross-dataset generalization in complex environments [21]. Different from the above studies that focus on general animal detection, face recognition, keypoint localization, or tracking, this study focuses on lightweight dairy cow detection under complex barn conditions, especially multi-scale appearance, occlusion, and model efficiency.

Despite these advances, a common limitation persists: improving detection accuracy under complex conditions typically comes at the cost of increased model complexity, making it difficult to simultaneously achieve high performance and efficient real-time deployment [22,23]. Although researchers have explored multi-scale feature fusion strategies to improve representation capability and lightweight convolution designs to lower computational cost, the accuracy–efficiency trade-off remains inadequately resolved for practical livestock monitoring scenarios.

To address this gap, this paper proposes DMFRNet, a model for dairy cow detection with three core contributions. First, the C3K2-DIMB module is designed, integrating dynamic inception depthwise convolution with data-driven kernel weighting for efficient multi-scale feature extraction. Second, the parameter-free SimAM attention mechanism is introduced to enhance feature discrimination through energy-based neuron importance evaluation. Third, a lightweight shared convolution detection head (LSCDH) with Group Normalization is constructed to reduce parameter count while maintaining detection performance. Experiments on the CBVD-5 dataset confirm the effectiveness of the collaborative optimization among the three modules.

## 2. Materials and Methods

### 2.1. Datasets

This study primarily employs CBVD-5 (Cow Behavior Video Dataset-5) as the base dataset, which was constructed and publicly released by Li et al. [24]. To ensure annotation quality, a rigorous cleaning process was applied to the original data: images with annotation omissions, duplicate annotations, severe occlusion, or low quality were removed, ultimately retaining 3432 valid annotated images.

To further enhance the model’s robustness under low-light conditions, 273 nighttime cattle images were selected and extracted from the Dairy Cow dataset on the Kaggle platform [25]. This dataset complements CBVD-5 in terms of cattle breeds and farming environments, effectively improving the scene diversity of the training data. After integration, a total of 3705 original images were obtained, which were expanded to 10,322 through data augmentation, providing a sufficient data foundation for deep learning model training and validation.

### 2.2. Data Processing

Deep learning model training imposes high demands on data scale and diversity; a single original dataset cannot support effective generalization under varying illumination, viewpoints, and occlusion conditions. Therefore, systematic data augmentation preprocessing was applied to the original images when constructing the experimental dataset to enhance model robustness and generalization capability.

**Figure 1:**
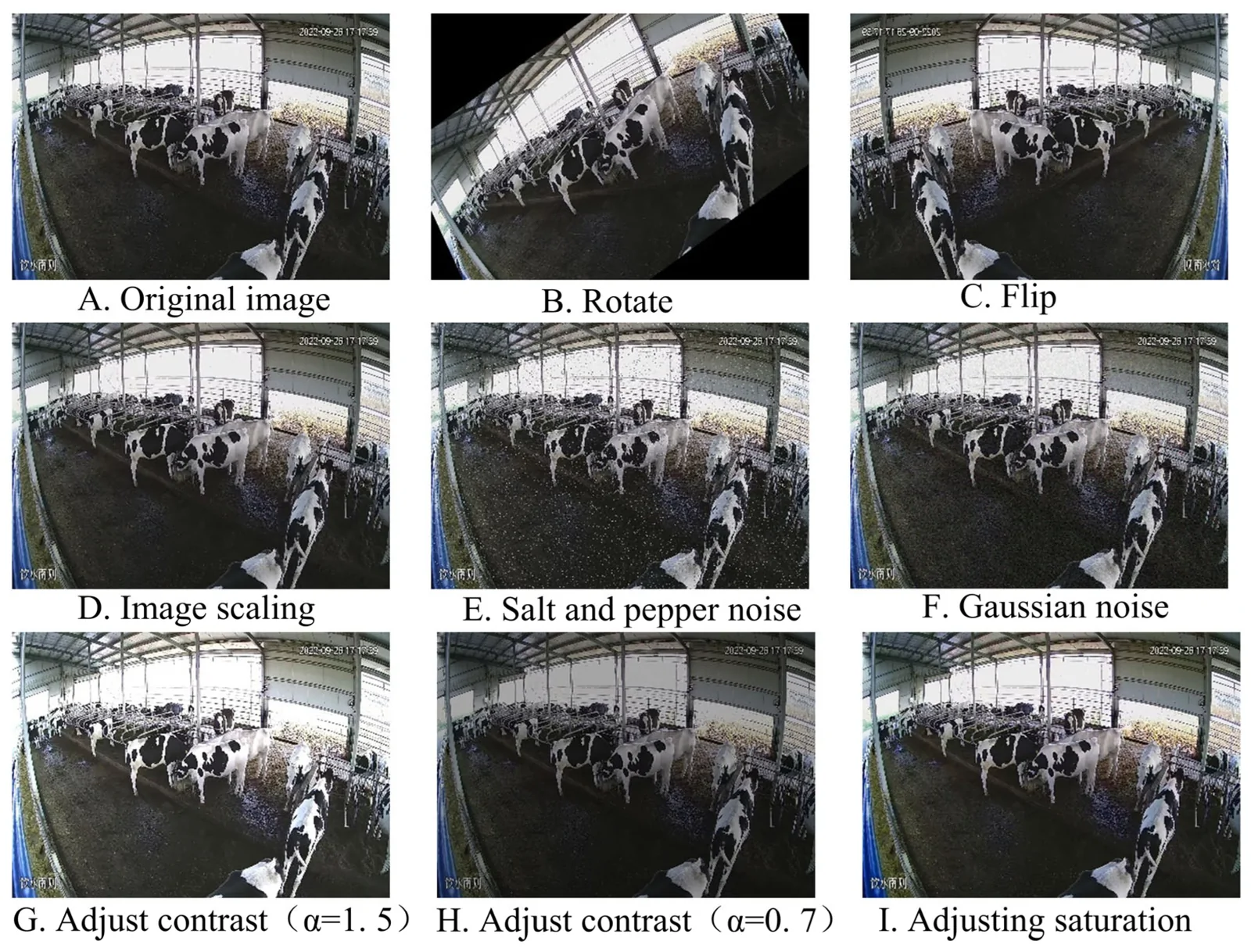
Illustration of data augmentation effects.

The adopted data augmentation strategy covers two major categories of operations: geometric transformations and pixel-level perturbations. Geometric transformations include random rotation, horizontal flipping, and image scaling, used to simulate cattle appearance variations under different shooting angles and distances. Pixel-level perturbations include image contrast adjustment, saturation adjustment, Gaussian noise injection, and salt-and-pepper noise injection, used to simulate practical scene disturbances such as varying illumination conditions, image quality degradation, and sensor noise. Examples of each augmentation operation are shown in Figure 1.

The dataset split was conducted at the original-image group level to prevent information leakage among subsets. Although data augmentation was performed before generating the final expanded dataset, each original image and all augmented variants derived from it were assigned exclusively to the same subset. The final dataset was divided into training, validation, and test sets at a ratio of 6:2:2. Therefore, no augmented variant derived from a training image appeared in the validation or test set, avoiding potential overestimation of model performance.

### 2.3. DMFRNet Architecture

This paper adopts YOLO11 as the baseline architecture [26]. YOLO11 consists of three components: Backbone, Neck, and Head. The Backbone is responsible for feature extraction, the Neck for multi-scale feature fusion, and the Head for object classification and localization. Compared with its predecessor YOLOv8, YOLO11 introduces the C3K2 structure and C2PSA module in the backbone network, enhancing feature representation capability through multi-stage convolution stacking and multi-head self-attention mechanisms. However, in cattle detection scenarios characterized by highly similar appearances, severe mutual occlusion, and significant scale variations, the fixed convolution kernel structure of C3K2 still exhibits insufficient adaptability to the aforementioned complex scenarios, and the decoupled design of the detection head introduces unnecessary parameter redundancy. Based on the above detection challenges, DMFRNet was designed to improve multi-scale feature extraction, semantic feature discrimination, and detection-head efficiency.

**Figure 2:**
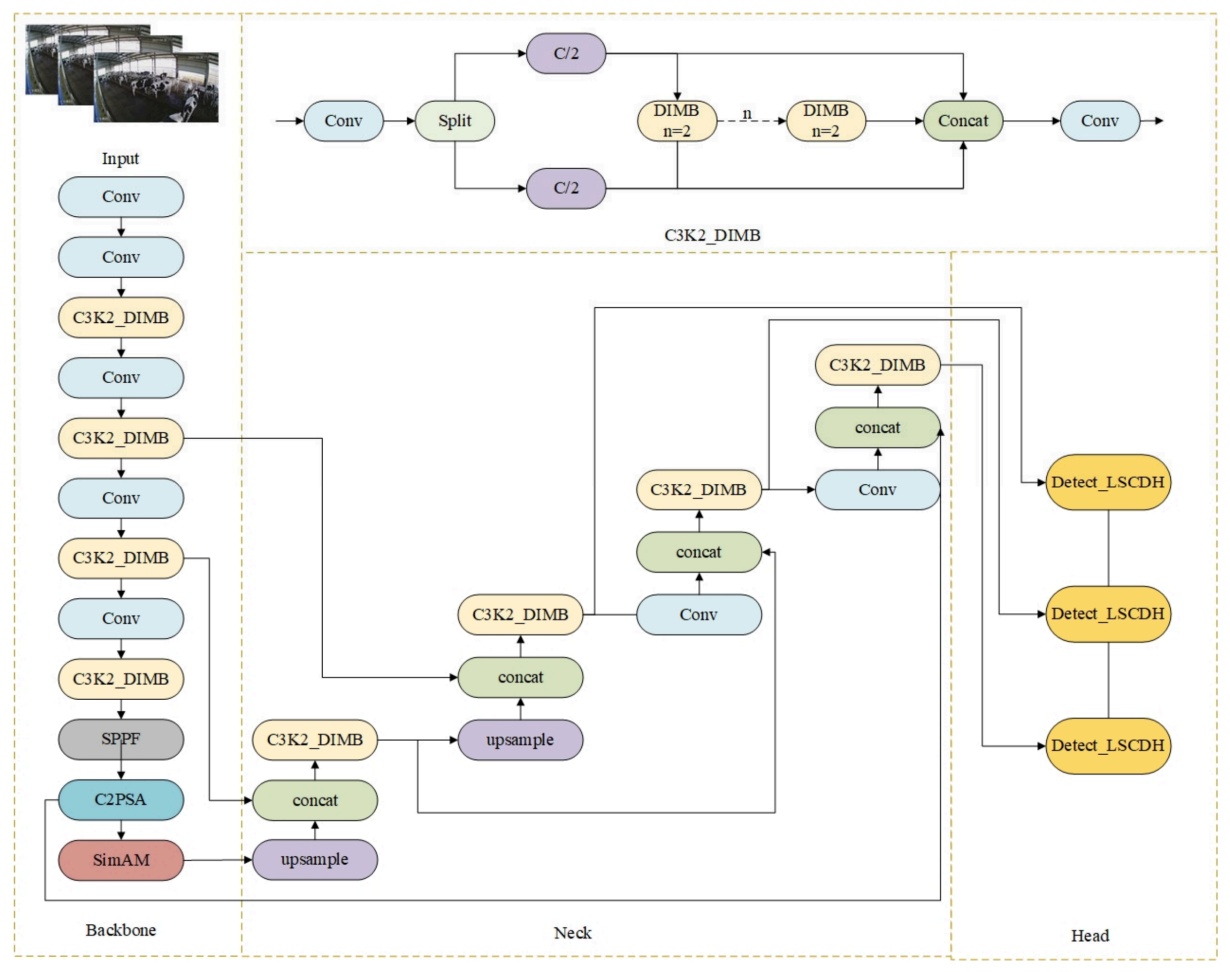
Schematic diagram of the DMFRNet architecture.

To address these issues, this paper proposes DMFRNet, whose overall architecture is illustrated in Figure 2. At the feature extraction level, the original C3K2 module is replaced with C3K2-DIMB, which adaptively modulates the contributions of multi-branch features through dynamic convolution kernel weights, enhancing the modeling capability for multi-scale variable objects. At the feature refinement level, the SimAM parameter-free attention mechanism is embedded after the SPPF layer at the end of the Backbone to strengthen the discriminability of high-level semantic features without introducing additional parameters, improving model robustness under illumination variation and occlusion conditions. At the detection output level, the original decoupled detection head is replaced with the lightweight shared convolution detection head, LSCDH, which shares convolution weights across scale branches and introduces a Scale Layer, effectively compressing parameter count and computational cost while maintaining multi-scale detection capability.

### 2.4. C3K2-DIMB

In ranch monitoring scenarios, dairy cows exhibit significant scale diversity—from distant small-sized individuals to nearby large-bodied cattle—with feature distributions spanning different spatial ranges. The original C3K2 module in YOLO11 adopts a fixed convolution kernel structure, where the contributions of each branch to multi-scale features are static and lack the ability to adaptively adjust according to input content.

**Figure 3:**
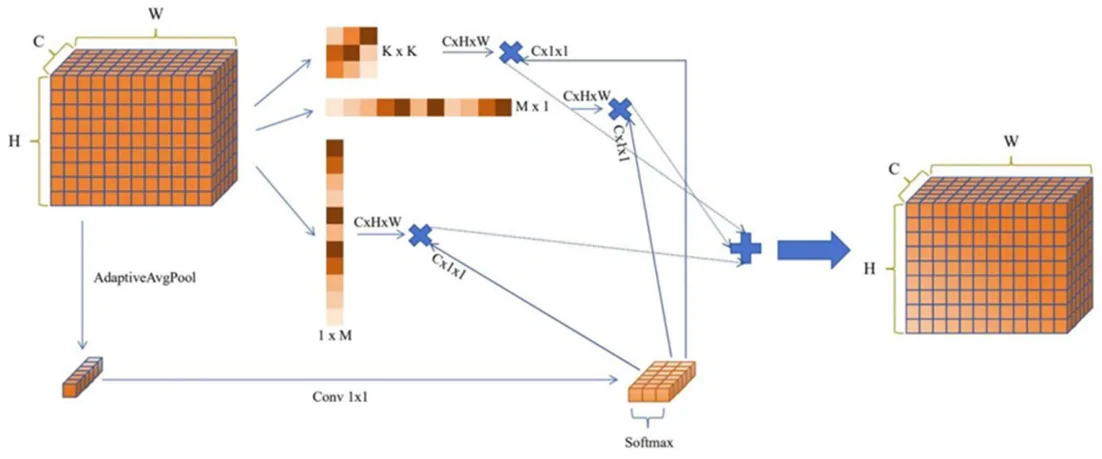
DIDWConv2d module.

To address this, this paper designs the C3K2-DIMB module, whose core is the Dynamic Incidence Mixer Block (DIMB), inspired by InceptionNeXt [27] and TransNeXt [28]. The structure of DIMB is shown in Figure 3. Following the MetaFormer paradigm, it consists of two cascaded stages: a DynamicInceptionMixer and a ConvolutionalGLU, responsible for spatial mixing and channel mixing respectively, with learnable Layer Scale factors introduced to stabilize training.

The workflow of the DynamicInceptionMixer is as follows. The input features are first equally split into two groups along the channel dimension, and each group is fed into a DIDWConv2d module. Within each unit, three depthwise convolution branches are deployed in parallel—a standard square kernel (3 × 3), a horizontal band kernel (1 × K), and a vertical band kernel (K × 1, where K = 3 × kernel_size + 2)—to capture spatial contexts of different shapes and ranges. Unlike fixed convolutions, this unit generates three sets of dynamic weights from the input features through adaptive average pooling and a 1 × 1 convolution. After Softmax normalization, the outputs of the three branches are weighted and fused, thereby realizing a dynamic modulation mechanism for convolution kernel weights. The core advantage of this design is that the network can automatically adjust the spatial modeling intensity along each direction according to the actual scale, pose, and background complexity of dairy cows in the current input.

C3K2-DIMB retains the cross-stage partial connection structure of C3K2 while replacing the internal feature transformation unit with DIMB. Compared with the original C3K2, this module achieves significantly improved multi-scale feature adaptability at a marginal increase in parameter count. While C3K2-DIMB enhances multi-scale spatial feature extraction, complex barn scenes also require stronger semantic discrimination under occlusion and low-contrast conditions. Therefore, SimAM is introduced for feature refinement.

### 2.5. SimAM

In cattle detection, factors such as severe illumination variation, mutual occlusion among objects, and high texture similarity between cattle and background lead to insufficient feature discriminability for certain objects, making them prone to missed detection or misclassification. Traditional attention mechanisms such as SE [29] and CBAM [30] enhance feature discriminability by introducing additional learnable parameters. However, channel attention and spatial attention are processed independently, limiting cross-dimensional interaction capability and increasing the parameter count.

**Figure 4:**
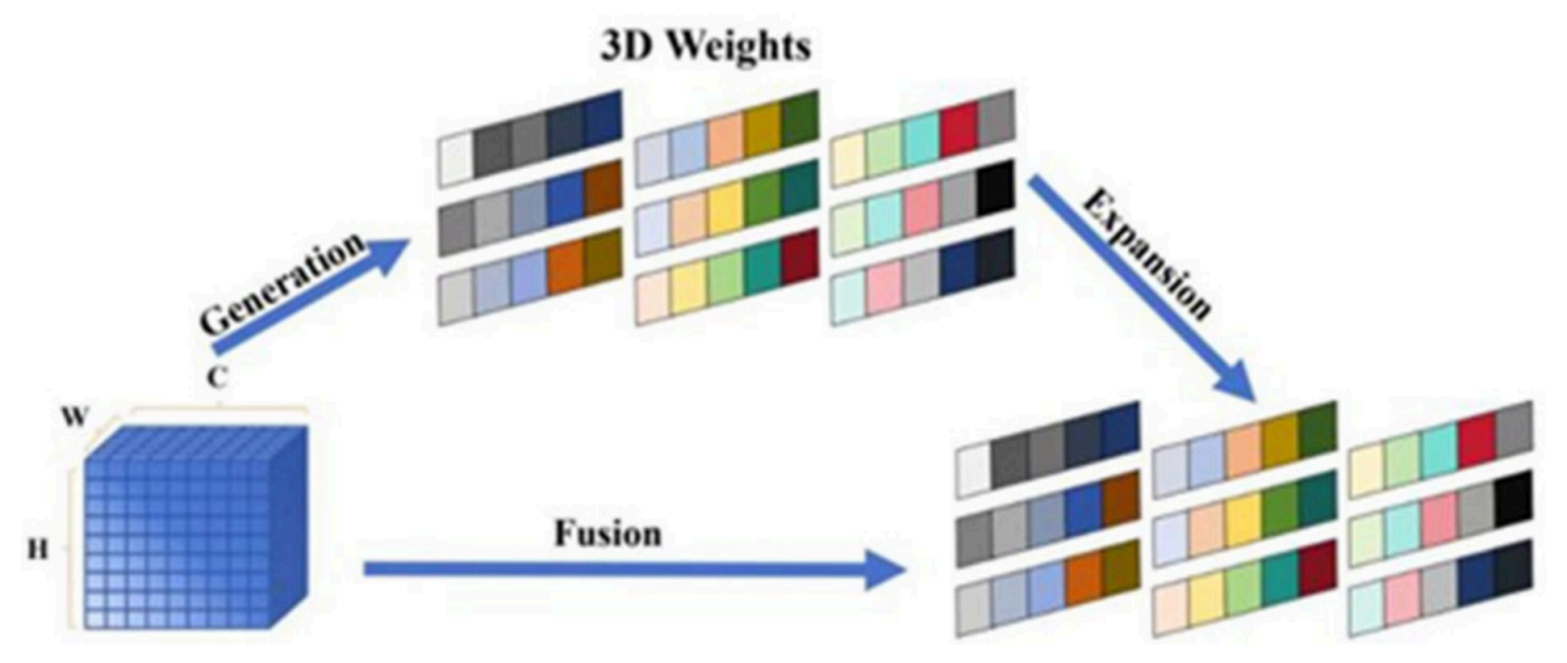
SimAM Attention Mechanism Architecture.

SimAM [31] is a lightweight attention mechanism grounded in a neuroscientific principle. This paper places it after the SPPF layer at the end of the Backbone to refine high-level semantic features. The structure of the SimAM attention mechanism is shown in Figure 4. Its core idea originates from a neuroscientific finding: neurons whose response patterns significantly differ from their surrounding neurons carry more discriminative information. SimAM evaluates the importance of each position in the feature map by computing an energy function: for each channel, the deviation of each pixel value from the channel mean is calculated and normalized by channel variance. Positions with larger deviations receive higher energy and greater attention weights. The energy function is defined as:

(1) $$e_{t}^{*}=\frac{4(\sigma^{2}+\lambda )}{{\left(t-\mu \right)}^{2}+2\sigma^{2}+2\lambda }$$

where *t* denotes the target pixel value, *μ* and *σ*^2^ denote the mean and variance of the channel, and *λ* is a balancing coefficient. The energy value is subsequently mapped to an attention weight via a Sigmoid function and multiplied element-wise with the original feature map to obtain the refined features.

SimAM introduces zero additional parameters, and its computational complexity is significantly lower than that of parameterized attention mechanisms such as CBAM and SE. In the proposed architecture, SimAM enables the high-level features output by the Backbone to acquire clearer semantic discriminability before entering the Neck for multi-scale fusion, contributing to improved detection performance under occlusion and low-contrast conditions. After improving feature extraction and semantic refinement, the detection head is further optimized to reduce redundant parameters while maintaining multi-scale prediction capability.

### 2.6. LSCDH

YOLO11 inherits the decoupled detection head design from YOLOv8, employing independent classification and regression convolution branches at three feature levels: P3, P4, and P5. This design avoids mutual interference between classification and regression tasks, but the independent parameterization of each level introduces substantial parameter redundancy, which is unfavorable for lightweight real-time detection.

**Figure 5:**
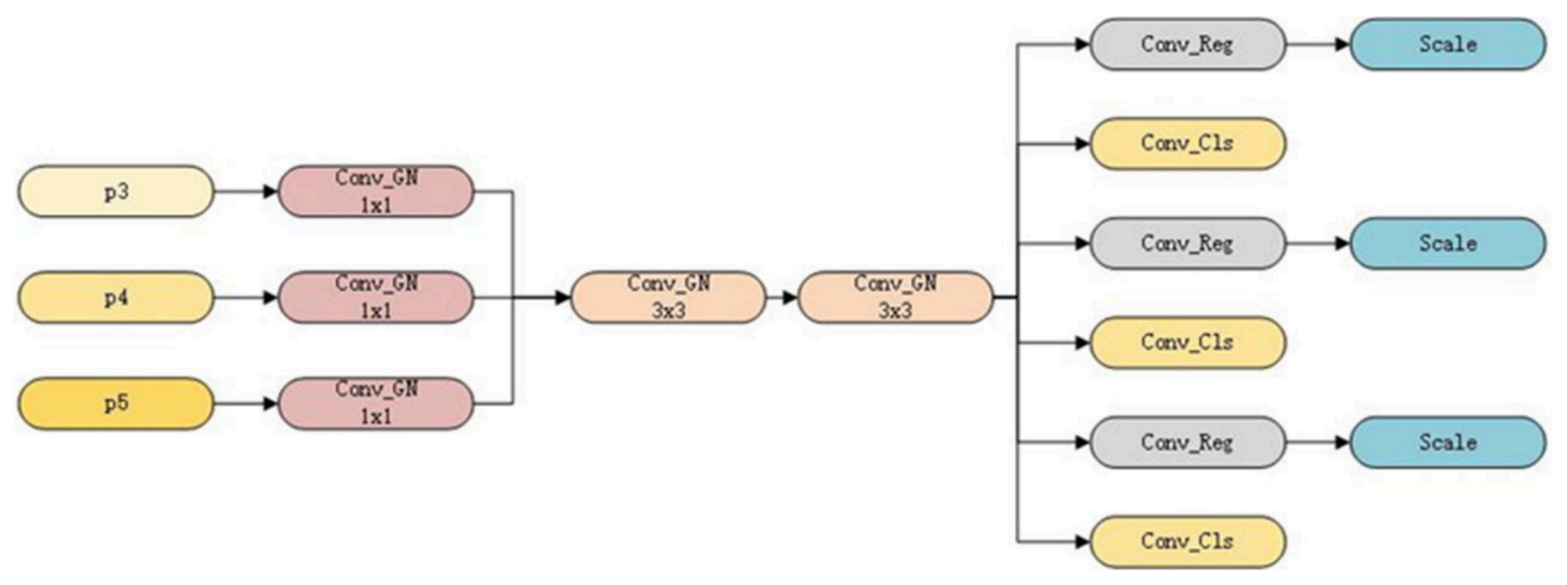
Architecture of the lightweight shared convolution detection head.

To address this, this paper designs a Lightweight Shared Convolution Detection Head, LSCDH, whose structure is illustrated in Figure 5. The lightweight nature of LSCDH stems from two core designs: cross-scale shared convolution and scale-adaptive adjustment.

First, multi-scale feature maps (P3, P4, P5) from the Neck each undergo a 1 × 1 convolution with GroupNorm for preliminary transformation, unifying features with different channel counts to the same hidden dimension. Subsequently, features from all scales share a cascaded set of convolutions—first a 3 × 3 channel-wise Conv_GN for spatial context aggregation, followed by a 1 × 1 Conv_GN for inter-channel information fusion. Since feature maps across the three detection scales share the same convolution weights, the parameter count of LSCDH is substantially reduced compared with independently parameterized decoupled heads.

Second, considering that different detection scales exhibit distinct feature distributions for classification and localization tasks, LSCDH introduces a learnable Scale parameter for each scale after the shared convolutions. Initialized at 1.0, this parameter automatically learns the optimal scaling factor for each scale during training, performing per-scale adjustment on the outputs of the classification and regression branches. This compensates for the loss of scale adaptability caused by weight sharing without incurring significant additional computational overhead.

Finally, the shared 1 × 1 convolution branches output bounding box regression parameters and class probabilities, respectively. In summary, LSCDH effectively compresses the parameter count and computational cost of the detection head while preserving multi-scale detection capability.

## 3. Results

### 3.1. Experimental Setup

All experiments were conducted on a Windows 10 64-bit operating system with an Intel(R) i5-9400 CPU (2.9 GHz base frequency, 4.1 GHz turbo frequency) and an NVIDIA GeForce GTX 1050 Ti GPU. The software development environment was PyCharm 2024.1.4 (Build 241.14494.241), implemented using Python 3.9 and the PyTorch 2.0.1 deep learning framework. All input images were uniformly resized to 640 × 640 pixels. The training hyperparameter settings are shown in Table 1. The model was trained until convergence after multiple rounds of hyperparameter tuning.

### 3.2. Evaluation Metrics

This paper adopts the following metrics to comprehensively evaluate model performance. Precision (P), F1-score, and mean Average Precision (mAP) are used to assess detection accuracy. mAP50 denotes mAP at an IoU threshold of 0.5, while mAP50–95 denotes the average mAP over 10 IoU thresholds from 0.5 to 0.95 in increments of 0.05; the latter more rigorously reflects the model’s localization accuracy. Parameter count (Params), floating-point operations (FLOPs), and model file size (Model Size) are adopted to evaluate model complexity and computational cost.

### 3.3. Training Convergence and Detection Performance Analysis

**Figure 6:**
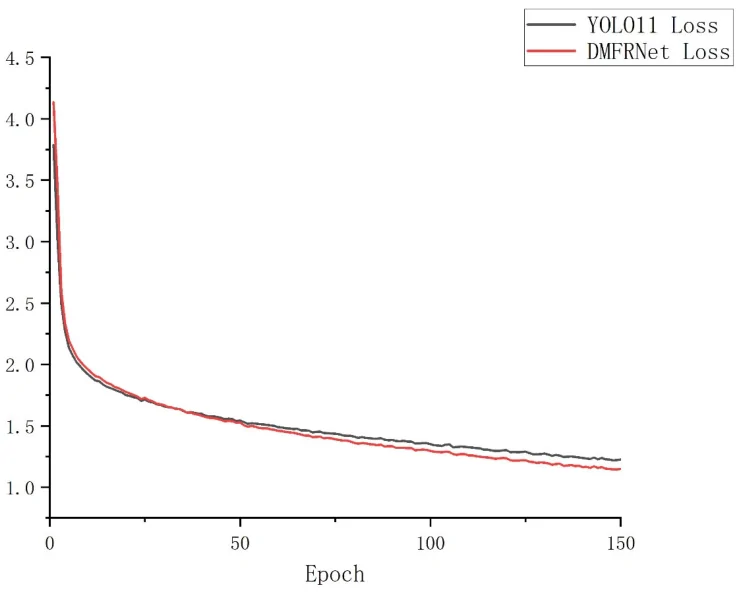
Training loss curves of DMFRNet and YOLO11.

To evaluate the training convergence characteristics of DMFRNet, the loss curves of DMFRNet and the baseline YOLO11 during training were compared, as shown in Figure 6. The loss drops sharply at the initial stage and then gradually stabilizes, exhibiting a pattern of rapid descent followed by slow convergence. DMFRNet recorded a loss of 4.14 at Epoch 1, slightly higher than YOLO11’s 3.79, but surpassed the baseline at Epoch 33, thereafter consistently maintaining a lower loss level. At the conclusion of training, the DMFRNet loss converged to 1.15, representing a 6.2% reduction compared with YOLO11’s 1.23. This indicates that its enhanced feature representation and optimized gradient propagation pathways effectively improve convergence quality and training stability.

In terms of detection performance, DMFRNet achieves a Precision of 93.49%, F1 of 89.84%, mAP50 of 93.85%, and mAP50–95 of 61.74% on the test set. The high mAP50 and mAP50–95 scores indicate that the model possesses robust object localization and recognition capability across different IoU thresholds. Regarding model complexity, DMFRNet has only 2.16 M parameters, 5.10 GFLOPs, and a model size of merely 4.5 MB, demonstrating a compact architectural design with low computational cost. The synergistic integration of C3K2-DIMB, SimAM, and LSCDH significantly improves detection accuracy while maintaining lightweight characteristics, supporting lightweight real-time detection potential.

### 3.4. Ablation Study

**Figure 7:**
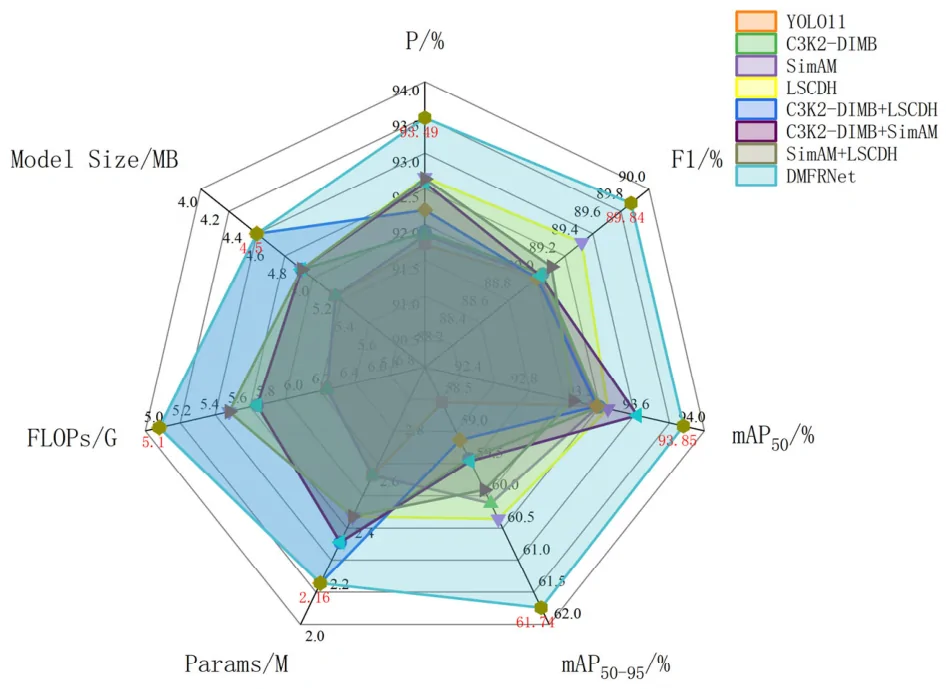
Radar chart showing the ablation performance of each module added to YOLO11. “C3K2-DIMB” indicates the replacement of the original C3K2 module with C3K2-DIMB; “SimAM” indicates the introduction of the SimAM attention mechanism; “LSCDH” indicates the replacement of the original detection head with LSCDH; “C3K2-DIMB + LSCDH”, “C3K2-DIMB + SimAM”, and “SimAM + LSCDH” indicate two-module combinations; “DMFRNet” represents the final model integrating C3K2-DIMB, SimAM, and LSCDH. A larger polygon area indicates better overall model performance.

A series of ablation experiments were conducted to evaluate the impact of each proposed improvement module on detection performance. All experiments were performed on the same dataset under consistent experimental conditions. The performance metrics are summarized in Table 2, and Figure 7 provides an intuitive comparison of parameter counts and accuracy across different module combinations. Note: A denotes the C3K2-DIMB lightweight backbone module, B denotes the SimAM parameter-free attention mechanism, and C denotes the LSCDH lightweight detection head.

The ablation study was conducted by incrementally integrating each proposed module into the baseline YOLO11 architecture. As shown in Table 2, replacing the C3K2 module in the backbone with C3K2-DIMB improved Precision, F1, mAP50, and mAP50–95 by 0.17, 0.05, 0.03, and 0.87 percentage points, respectively. The parameter count decreased from 2.58 M to 2.32 M, GFLOPs dropped from 6.30 to 5.80, and model size shrank from 5.2 MB to 4.9 MB. Introducing the SimAM attention mechanism improved Precision and F1 by 0.13 and 0.02 percentage points, respectively, and raised mAP50–95 by 1.57 percentage points to 60.12%, with the parameter count, FLOPs, and model size remaining identical to the baseline, demonstrating that SimAM achieves effective detection accuracy improvement without introducing additional parameters. Adopting the LSCDH detection head improved Precision by 0.94 percentage points, F1 by 0.40 percentage points, mAP50 by 0.09 percentage points, and mAP50–95 by 1.81 percentage points to 60.36%, while reducing FLOPs by 11.1% to 5.60, the parameter count to 2.42 M, and model size to 4.9 MB, achieving the largest accuracy gain among all single-module improvements. These single-module results indicate that the three improvements contribute through different mechanisms. C3K2-DIMB mainly improves multi-scale localization, because its dynamic multi-branch depthwise convolution can adaptively capture spatial patterns of cattle with different body sizes, poses, and viewing distances. SimAM improves mAP50–95 without increasing model complexity, suggesting that the parameter-free attention mechanism enhances discriminative high-level features under occlusion, low contrast, and complex backgrounds. LSCDH contributes mainly to detection-head compression and precision improvement, indicating that cross-scale shared convolution can reduce redundant head parameters while preserving useful multi-scale prediction capability.

To further justify the placement of SimAM after the SPPF layer, an additional position-wise ablation experiment was conducted. As shown in Table 3, placing SimAM at position C achieved the best performance among the three tested positions, with Precision, F1, mAP50, and mAP50–95 reaching 91.85%, 89.02%, 93.24%, and 60.12%, respectively. Compared with positions A and B, position C produced consistently higher values across all four accuracy metrics. This result indicates that applying SimAM after the SPPF layer is more effective for refining high-level semantic features before multi-scale feature fusion, thereby supporting the rationality of the proposed placement.

Multi-module combinations were further evaluated to assess synergistic effects. The combination of C3K2-DIMB and LSCDH further reduced the parameter count by 16.3% to 2.16 M, FLOPs by 19.0% to 5.10, and model size by 13.5% to 4.5 MB, achieving the strongest lightweight effect among all combinations; however, mAP50 improved by only 0.01 percentage points, and F1 remained at the baseline level. The combination of C3K2-DIMB and SimAM improved Precision by 0.86 percentage points and raised mAP50 by 0.30 percentage points to 93.52%, the best mAP50 among dual-module combinations, while maintaining the parameter count at 2.32 M; however, mAP50–95 improved by only 0.91 percentage points. The combination of SimAM and LSCDH improved Precision by 0.92 percentage points and F1 by 0.13 percentage points, achieving the best Precision among dual-module combinations; however, mAP50 experienced a slight decline of 0.16 percentage points, suggesting a degree of feature competition between SimAM and LSCDH. These dual-module results show that the effects of the three modules are not simply additive. The C3K2-DIMB + LSCDH combination achieves the strongest lightweight effect but limited accuracy improvement, indicating that simultaneous backbone and head compression requires sufficient feature refinement. The C3K2-DIMB + SimAM combination achieves the best mAP50 among dual-module combinations, suggesting that dynamic multi-scale feature extraction and attention-based semantic refinement are complementary. In contrast, the slight mAP50 decline in the SimAM + LSCDH combination suggests that attention refinement and shared detection-head prediction may introduce partial feature competition. Therefore, the three-module design of DMFRNet is necessary to balance multi-scale feature extraction, semantic refinement, and lightweight detection-head prediction.

Finally, the complete integration of all three modules—C3K2-DIMB, SimAM, and LSCDH—constitutes the proposed DMFRNet model. Relative to the baseline YOLO11, this configuration improved Precision by 1.77 percentage points to 93.49%, F1 by 0.84 percentage points to 89.84%, mAP50 by 0.63 percentage points to 93.85%, and mAP50–95 substantially by 3.19 percentage points to 61.74%, while simultaneously reducing the parameter count to 2.16 M, FLOPs to 5.10, and model size to 4.5 MB. These results indicate that DMFRNet provides a favorable accuracy–efficiency trade-off by jointly improving localization quality, reducing model complexity, and maintaining compact model size.

### 3.5. Comparison of C3K2 Improvement Schemes

To further justify the selection of C3K2-DIMB as the C3K2 improvement scheme, three C3K2 variants—C3K2-PFDConv [32], C3K2-DBlock [33], and C3K2-DIMB—were compared under identical experimental conditions. The results are presented in Table 4.

In terms of detection accuracy, C3K2-DIMB achieved the best results across all metrics: Precision of 91.89%, F1 of 89.05%, mAP50 of 93.25%, and mAP50–95 of 59.42%, comprehensively outperforming both C3K2-PFDConv and C3K2-DBlock. C3K2-PFDConv attained a Precision of 91.73% and mAP50 of 92.45%, which were 0.16 and 0.80 percentage points lower than C3K2-DIMB, respectively. C3K2-DBlock exhibited the poorest accuracy, with a Precision of only 90.26% and mAP50–95 of merely 56.23%, representing declines of 1.63 and 3.19 percentage points relative to C3K2-DIMB, respectively—a substantial degradation. In terms of model complexity, C3K2-PFDConv had the smallest parameter count and lowest FLOPs, but at the expense of accuracy loss. C3K2-DBlock, with 2.31 M parameters and 5.90 GFLOPs, demonstrated no competitive advantage in either accuracy or efficiency.

**Figure 8:**
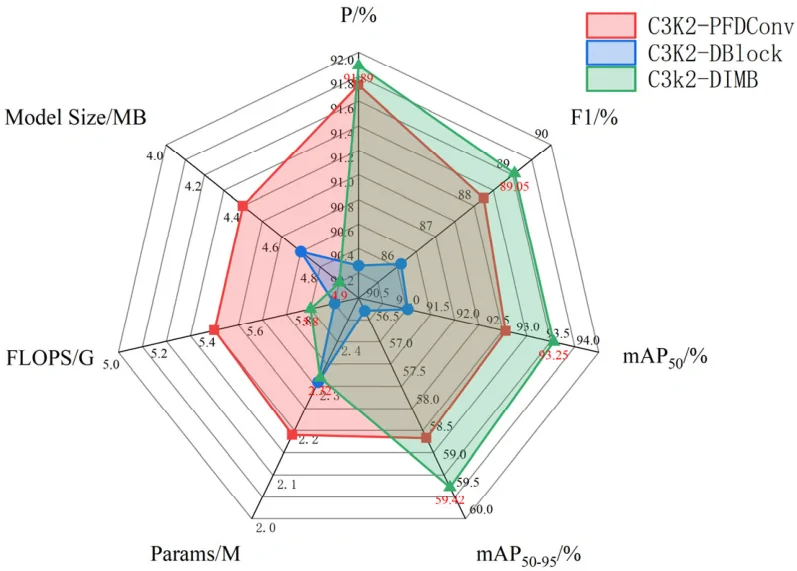
Radar chart comparing the performance of different C3K2 improvement schemes. “C3K2-PFDConv” indicates the replacement of the original C3K2 module with C3K2-PFDConv; “C3K2-DBlock” indicates the replacement of the original C3K2 module with C3K2-DBlock; “C3K2-DIMB” indicates the replacement of the original C3K2 module with the proposed C3K2-DIMB. A larger polygon area indicates better overall model performance.

In summary, C3K2-DIMB achieves stronger detection accuracy than the other C3K2 variants, with only a moderate increase in parameter count compared with C3K2-PFDConv, providing a favorable accuracy–efficiency trade-off. Figure 8 further visualizes the overall performance differences among the three C3K2 improvement schemes.

### 3.6. Comparative Experiment and Visualization Analysis

To comprehensively evaluate the detection performance of DMFRNet, the proposed model was quantitatively compared with YOLOv8n [34], YOLOv9t [35], YOLO11, YOLO12n [36], and Hyper-YOLO [37] under identical experimental conditions. The results are presented in Table 5.

As shown in Table 5, DMFRNet achieves the best Precision and mAP50–95 among all compared models, with its mAP50 second only to Hyper-YOLO. Relative to the baseline YOLO11, Precision improves by 1.77 percentage points and mAP50–95 by 3.19 percentage points. In terms of model complexity and inference efficiency, DMFRNet has only 2.16 M parameters, 5.10 GFLOPs, and a model size of 4.5 MB, while achieving 104.57 FPS under the same hardware environment and input-size setting. Although Hyper-YOLO is marginally superior in F1 and mAP50, DMFRNet achieves higher mAP50–95, fewer parameters, lower FLOPs, a smaller model size, and faster inference speed than Hyper-YOLO. These results indicate that DMFRNet does not rely on superiority in a single metric; instead, it provides a favorable accuracy–efficiency trade-off for lightweight dairy cow detection by jointly considering detection accuracy, localization quality, model complexity, storage cost, and inference speed.

**Figure 9:**
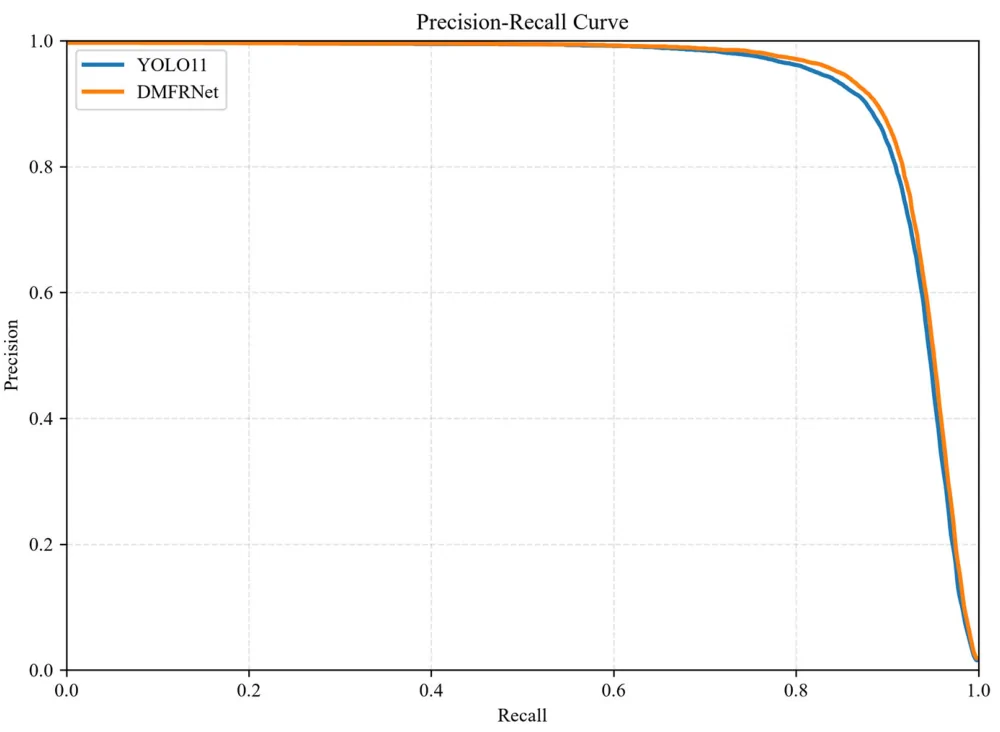
Precision-Recall curves of the baseline YOLO11 and the proposed DMFRNet.

To further visualize the performance improvement introduced by the proposed method, the Precision-Recall (PR) curves of YOLO11 and DMFRNet were plotted, as shown in Figure 9. Compared with the baseline YOLO11, DMFRNet maintains a higher precision level over a broad recall range, indicating improved detection stability after integrating the proposed modules.

**Figure 10:**
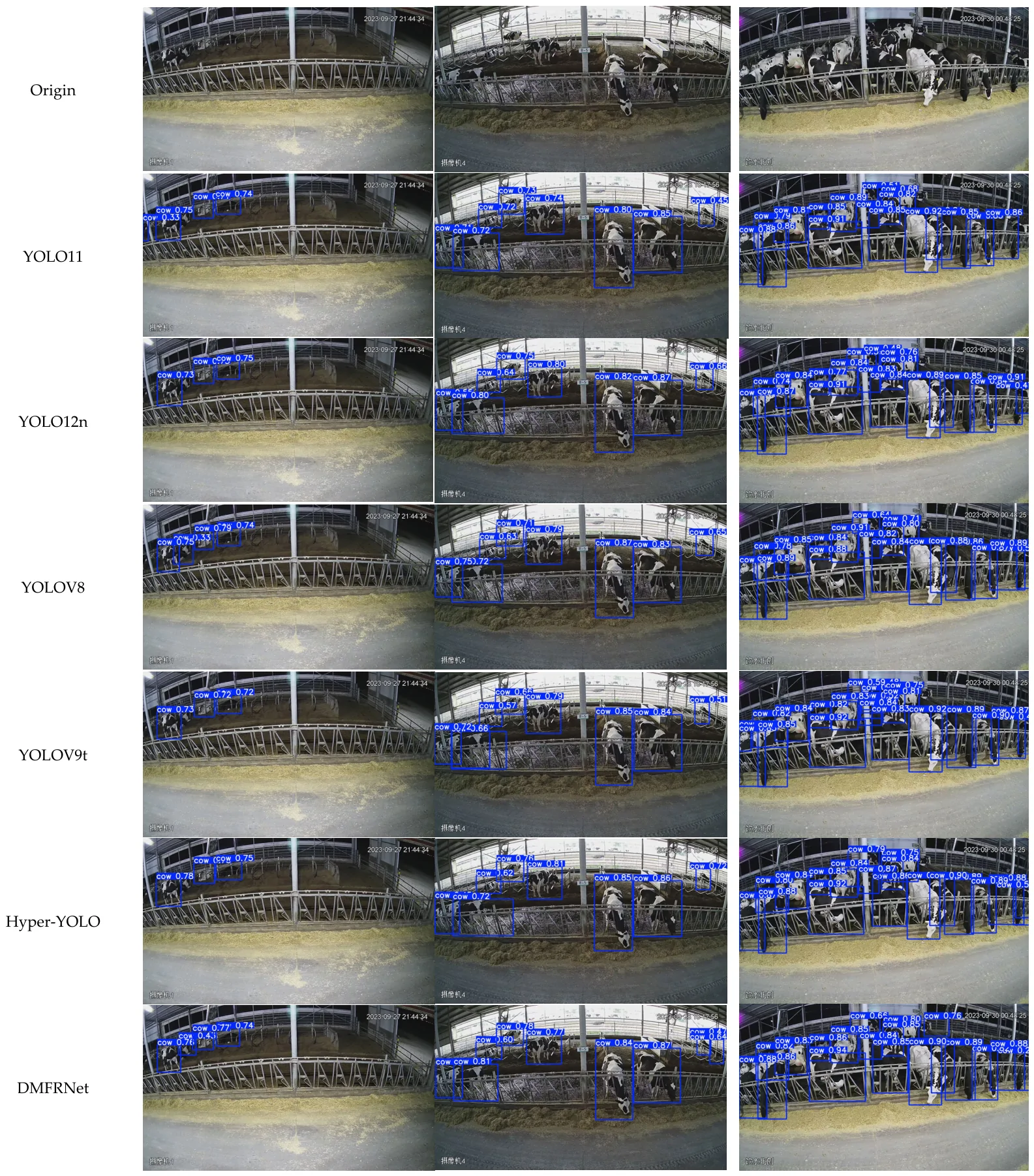
Visualization analysis of different models.

To intuitively verify the above quantitative conclusions, three representative scenarios—sparse objects, medium-density complex backgrounds, and high-density crowded scenes—were selected from the test set for visualization comparison. The results are shown in Figure 10. In the sparse scenario, YOLOv9t, YOLO12n, and Hyper-YOLO all missed a small-sized cow in the left-center region of the image, likely because the object is small with low contrast against the surrounding background texture, and the aforementioned models had insufficient feature extraction capability for it. YOLO11 and DMFRNet both successfully detected this cow, correctly identifying all four objects. Additionally, YOLOv9t and YOLO11 falsely detected truncated cattle body parts near the left image boundary as independent objects, producing extra false positive boxes, reflecting insufficient contextual reasoning for boundary truncation regions. DMFRNet exhibited no such false detections in this scenario. In the medium-density complex background scenario, YOLO11 and YOLOv9t produced numerous false positive boxes in the left and right edge truncation regions, likely because edge truncation led to incomplete cattle features that were fragmentarily recognized as multiple objects by the models. YOLOv8n and YOLO12n also exhibited a certain degree of edge false positives. DMFRNet had the fewest false detections in this scenario, with the cleanest boundary region processing, indicating superior holistic discriminative ability for truncated cattle. In the high-density crowded scenario, DMFRNet correctly detected all cattle, with no missed detections of distant small cattle and no redundant boxes on nearby large cattle. YOLOv9t produced multiple highly overlapping detection boxes for the same large cattle, exhibiting duplicate detection, likely because high feature similarity among dense objects caused the detection head to generate redundant candidate boxes. Hyper-YOLO failed to effectively identify some small-sized cattle in distant regions, possibly due to insufficient response intensity to extremely small-scale objects. YOLO11 and YOLO12n exhibited relatively more false positives in small-object and boundary regions, similarly affected by feature fragmentation and boundary truncation. Across the three representative scenarios, DMFRNet showed stable performance in missed detection control and false positive suppression, which was consistent with its quantitative advantages in Precision and mAP50–95.

## 4. Discussion

The above experimental results demonstrate the quantitative performance of DMFRNet; this section further discusses the effectiveness of the proposed modules and the remaining limitations.

### 4.1. Effectiveness Analysis of Improvements

The ablation study results demonstrate that the three modules—C3K2-DIMB, SimAM, and LSCDH—each contribute positively to model performance from three dimensions: feature extraction, feature refinement, and detection output. C3K2-DIMB improves mAP50–95 by 0.87 percentage points while reducing the parameter count by 10.1%; SimAM improves mAP50–95 by 1.57 percentage points without introducing additional parameters; and LSCDH improves Precision by 0.94 percentage points while achieving 6.2% parameter compression. Notably, the mAP50–95 gain from the full integration of all three modules is smaller than the sum of the individual module gains, and the mAP50 of the SimAM + LSCDH combination experienced a slight decline, suggesting a degree of feature redundancy and competition among modules. Nevertheless, the three-module synergy still provides the most favorable accuracy–efficiency trade-off among the ablation variants, indicating that the complementarity among C3K2-DIMB, SimAM, and LSCDH outweighs their potential feature competition. In terms of the accuracy–efficiency trade-off, DMFRNet has only 2.16 M parameters and 5.10 GFLOPs, the lowest among all compared models. Although Hyper-YOLO is marginally superior in mAP50 and F1, its FLOPs are 1.86 times those of DMFRNet. DMFRNet achieves the best Precision and mAP50–95 while maintaining the lowest computational cost, validating the advantages of the synergistic module design for resource-constrained monitoring scenarios.

It should also be noted that the performance gains of DMFRNet over the YOLO11 baseline are not uniform across all metrics. The improvements in mAP50 and F1 are relatively modest, increasing by 0.63 and 0.84 percentage points, respectively. However, mAP50–95 improves by 3.19 percentage points, suggesting that DMFRNet provides a more noticeable improvement in localization quality under stricter IoU thresholds. In addition, this improvement is achieved with fewer parameters, lower FLOPs, a smaller model size, and real-time inference speed. Therefore, the practical value of DMFRNet lies not in a large improvement in a single accuracy metric, but in its combined improvement in localization quality and model efficiency. Since repeated training experiments were not conducted in this study, statistical significance, confidence intervals, and standard deviations are not reported. This limitation has been acknowledged, and multi-run evaluation will be considered in future work to further assess the robustness of the observed performance gains.

### 4.2. Visualization Analysis and Limitations

The visualization comparison across three representative scenarios further reveals the sources of DMFRNet’s performance advantages. The ability to detect low-contrast small objects in sparse scenes is attributed to the dynamic multi-scale enhancement of C3K2-DIMB and the discriminability refinement of SimAM. The absence of missed detections and redundant boxes in high-density scenes is attributed to the favorable scale discrimination capability of LSCDH for multi-scale object localization. The suppression of false positives in boundary truncation regions is attributed to the enhanced global context modeling from three-module synergy. This study still has the following limitations. The experimental dataset is relatively concentrated in terms of cattle breeds and farming environments, and the model’s cross-scenario generalization capability requires further validation. Although FPS was evaluated under the same hardware environment and input-size setting, real-device inference speed testing on embedded or resource-constrained hardware has not yet been conducted. Therefore, latency stability and hardware adaptability still require further validation. The visualization analysis remains qualitative, without the introduction of interpretability tools such as Grad-CAM for quantitative attribution analysis. Future work will focus on four directions: cross-domain dataset expansion, hardware-aware inference optimization and validation in resource-constrained environments, temporal behavioral tracking and analysis, and feature visualization-based attribution to further reveal the mechanistic sources of accuracy gains.

## 5. Conclusions

This paper addresses three major challenges in cattle detection within complex barn environments—highly similar appearances, severe occlusion, and significant multi-scale variations—by proposing a lightweight cattle detection model, DMFRNet, with YOLO11 as the baseline. The model designs the C3K2-DIMB module, replacing fixed convolution kernels with dynamic inception depthwise convolutions to achieve dynamic weighted fusion of multi-branch features, thereby enhancing multi-scale object feature modeling capability. The SimAM parameter-free attention mechanism is introduced to refine high-level semantic features from the Backbone without introducing additional parameters, improving object discriminability under occlusion and low-contrast conditions. The LSCDH lightweight shared convolution detection head is constructed, which effectively compresses detection head parameter redundancy through cross-scale shared convolution weights and scale-adaptive adjustment while preserving multi-scale detection capability.

Experimental results on the CBVD-5 and Dairy Cow datasets demonstrate that DMFRNet achieves a Precision of 93.49%, F1 of 89.84%, mAP50 of 93.85%, and mAP50–95 of 61.74%, with only 2.16 M parameters, 5.10 GFLOPs, and a model size of merely 4.5 MB. The ablation study validates the effectiveness and synergistic contributions of each improvement module. Quantitative comparison and visualization analysis against YOLOv8n, YOLOv9t, YOLO11, YOLO12n, and Hyper-YOLO further demonstrate that DMFRNet provides a favorable accuracy–efficiency trade-off between detection accuracy and model efficiency, offering a viable lightweight solution for accurate and efficient cattle detection in complex barn environments.

## Figures and Tables

**Table 1:** Training hyperparameter settings.

Parameter	Value
Batch Size	16
Epochs	150
Lr_0_	0.01
Lrf	0.01
Momentum	0.937
Weight Decay	0.0005
Optimizer	SGD
Input Size	640 × 640

**Table 2:** Ablation study results. A denotes C3K2-DIMB, B denotes SimAM, and C denotes LSCDH.

A	B	C	P/%	F1/%	mAP50/%	mAP50–95/%	Params/M	FLOPs/G	Size/MB
			91.72	89.00	93.22	58.55	2.58	6.3	5.2
✓			91.89	89.05	93.25	59.42	2.32	5.8	4.9
	✓		91.85	89.02	93.24	60.12	2.58	6.3	5.2
		✓	92.66	89.40	93.31	60.36	2.42	5.6	4.9
✓		✓	92.20	89.00	93.23	59.13	2.16	5.1	4.5
✓	✓		92.58	89.03	93.52	59.46	2.32	5.8	4.9
	✓	✓	92.64	89.13	93.06	59.91	2.42	5.6	4.9
✓	✓	✓	93.49	89.84	93.85	61.74	2.16	5.1	4.5

**Table 3:** Ablation results of different SimAM placement strategies. A denotes SimAM before the SPPF layer, B denotes SimAM after the Neck and before the detection head, and C denotes SimAM after the SPPF layer.

Position	P/%	F1/%	mAP50/%	mAP50–95/%
A	91.70	88.77	92.87	59.85
B	91.52	88.33	93.11	59.61
C	91.85	89.02	93.24	60.12

**Table 4:** Performance comparison of different C3K2 improvement schemes.

Model	P/%	F1/%	mAP50/%	mAP50–95/%	Params/M	FLOPs/G	Model Size/MB
C3K2-PFDConv	91.73	88.25	92.45	58.54	2.19	5.4	4.4
C3K2-DBlock	90.26	86.10	90.82	56.23	2.31	5.9	4.7
C3K2-DIMB	91.89	89.05	93.25	59.42	2.32	5.8	4.9

**Table 5:** Performance comparison of different models.

Model	P/%	F1/%	mAP50/%	mAP50–95/%	Params/M	FLOPs/G	Model Size/MB	FPS
YOLO11	91.72	89.00	93.22	58.55	2.58	6.3	5.2	152.40
YOLOv8n	91.89	89.30	93.30	60.35	2.68	6.8	5.4	127.01
YOLOv9t	91.91	88.73	93.04	59.88	1.73	6.4	4.0	104.03
YOLO12n	90.72	89.25	93.08	60.11	2.51	5.8	5.2	100.76
Hyper-YOLO	92.89	90.26	93.99	60.07	3.62	9.5	7.3	78.97
DMFRNet	93.49	89.84	93.85	61.74	2.16	5.1	4.5	104.57

## Data Availability

The CBVD-5 dataset is publicly available at https://www.kaggle.com/datasets/fandaoerji/cbvd-5cow-behavior-video-dataset (accessed on 22 July 2026). The Dairy Cow dataset is available at https://www.kaggle.com/datasets/twisdu/dairy-cow (accessed on 22 July 2026). The source code presented in this study is available on request from the corresponding author.

## Abbreviations

The following abbreviations are used in this manuscript:

YOLO	You Only Look Once
DMFRNet	Dynamic Multi-scale Feature Reweighting Network
C3K2-DIMB	C3K2 Dynamic Incidence Mixer Block
DIMB	Dynamic Incidence Mixer Block
SimAM	Simple, Parameter-Free Attention Module
LSCDH	Lightweight Shared Convolution Detection Head
CBVD-5	Cow Behavior Video Dataset-5
DIDWConv2d module	DynamicInceptionDWConv2d module
IoU	Intersection over Union
FLOPs	Floating Point Operations
GFLOPs	Giga Floating Point Operations
FPS	Frames Per Second
SPPF	Spatial Pyramid Pooling-Fast
mAP	mean Average Precision
mAP50	mean Average Precision at an IoU threshold of 0.5
mAP50–95	mean Average Precision averaged over IoU thresholds from 0.5 to 0.95
Params	Parameter count
F1	F1-score
SE	Squeeze-and-Excitation
CBAM	Convolutional Block Attention Module
CNN	Convolutional Neural Network
CPU	Central Processing Unit
GPU	Graphics Processing Unit
SGD	Stochastic Gradient Descent
